# Identifying Facilitators and Inhibitors of Shared Understanding: An Ethnography of Diagnosis Communication in Acute Medical Settings

**DOI:** 10.1111/hex.14180

**Published:** 2024-08-24

**Authors:** Caitríona Cox, Thea Hatfield, Janet Willars, Zoë Fritz

**Affiliations:** ^1^ The Healthcare Improvement Studies Institute Cambridge UK

**Keywords:** communication, diagnosis, ethnography, shared understanding

## Abstract

**Background and Aims:**

Communication is important in determining how patients understand the diagnostic process. Empirical studies involving direct observation of communication within diagnostic processes are relatively limited. This ethnographic study aimed to identify communicative practices facilitating or inhibiting shared understanding between patients and doctors in UK acute secondary care settings.

**Methods:**

Data were collected in acute medical sectors of three English hospitals. Researchers observed doctors as they assessed patients; semistructured interviews were undertaken with doctors and patients directly afterwards. Patients were also interviewed 2–4 weeks later. Case studies of individual encounters (consisting of these interviews and observational notes) were created, and were cross‐examined by an interdisciplinary team to identify divergence and convergence between doctors' and patients' narratives. These data were analysed thematically.

**Results:**

We conducted 228 h of observation, 24 doctor interviews, 32 patient interviews and 15 patient follow‐up interviews. Doctors varied in their communication. Patient diagnostic understanding was sometimes misaligned with that of their doctors; interviews revealed that they often made incorrect assumptions to make sense of the fragmented information received. Thematic analysis identified communicative practices that seemed to facilitate, or inhibit, shared diagnostic understanding between patient and doctor, revealing three themes: (1) communicating what has been understood from the medical record, (2) sharing the thought process and diagnostic reasoning and (3) closing the loop and discharge communication. Shared understanding was best fostered by clear communication about the diagnostic process, what had already been done and what was achievable in acute settings. Written information presents an underutilised tool in such communication.

**Conclusions:**

In UK acute secondary settings, the provision of more information about the diagnostic process often fostered shared understanding between doctor and patient, helping to minimise the confusion and dissatisfaction that can result from misaligned expectations or conclusions about the diagnosis, and the uncertainty therein.

**Patient/Public Contribution:**

A patient and public involvement group (of a range of ages and backgrounds) was consulted. They contributed to the design of the protocol, including the timing of interviews, the acceptability of a follow‐up telephone interview, the development of the interview guides and the participant information sheets.

## 1 Introduction

Diagnosis has been described as a ‘ritual of disclosure’ [1]: throughout the diagnostic process, doctors must decide what information to impart to their patients. The process can be considered a journey along that both patient and doctor travel [2, 3, 4]; communication plays a key role in determining the extent to which they travel this journey together or apart [5]. Traditional conceptualisations—whereby a doctor gathers information before finally ‘delivering the diagnosis’ at the end of the clinical encounter—do not reflect the modern diagnostic process [3, 4]. As Rising et al. more accurately describe, diagnosis is ‘an iterative cycle of information gathering, interpretation and synthesis to develop a set of working diagnoses and ultimately a final diagnosis’ [6].

Communication can be particularly difficult in acute settings: challenges include time constraints, a chaotic and noisy environment, frequent interruptions, transient interactions where healthcare professionals lack an existing therapeutic relationship with the patient and patient distress in the context of acute illness [7, 8, 9, 10, 11]. That patients often have a poor understanding of their diagnosis after discharge from acute settings has been widely demonstrated [12, 13, 14, 15, 16, 17, 18, 19, 20].

Ineffective communication about diagnosis can result in doctor–patient misunderstandings, with potentially serious consequences. Several studies have found that poor patient understanding (of their diagnosis or the rationale for follow‐up) is associated with decreased concordance with discharge instructions and follow‐up [21, 22, 23, 24]. Misalignments between doctor and patients can negatively impact upon timely diagnosis: if patients do not understand their doctors' concerns surrounding suspected cancer symptoms [25] or if they do not appreciate the provisional nature of a working diagnosis [26]. Poor communication can also have legal and ethical consequences: patient autonomy may be undermined by a failure to communicate material information regarding diagnosis. Indeed, legal scholars have questioned whether the *Montgomery* ruling [27] (which established UK legal standards to inform patients about alternative treatments when consenting) might also give rise to a duty for doctors to not just disclose the likely diagnosis but also *reasonable alternate* diagnoses [28].

Despite its importance, communication within the diagnostic process has been relatively underexplored empirically [29]. A few studies have harnessed lived patient/caregiver experiences (e.g., a study that analysed narratives submitted by patients/caregivers, highlighting communication issues contributing to diagnostic errors) [30]. More broadly, interview studies have explored patient experiences of diagnosis for a range of conditions (including MND [31], amyloidosis [32], cancer [26] and gout [33]). However, few of these studies specifically explored how doctors communicated throughout the diagnostic process; moreover, the generalisability of research examining very specific conditions to diagnosis more broadly is questionable.

Much of the existing research has been conducted retrospectively, and studies involving participant recall of events are vulnerable to hindsight bias [34]. Research involving direct observation of diagnosis formation/communication in real time avoids such bias. A small number of studies have used ethnographic techniques to directly observe diagnostic processes as they occur [11, 20, 35, 36]; others have video‐ or audio‐taped encounters to study diagnostic communication [25, 37]. These studies suggest that information provision is often poor, particularly about possible diagnoses and investigations: in a US study, emergency department doctors failed to convey results of blood tests in 42.9% of encounters and failed to relate a diagnosis (or lack of diagnosis) to patients in 23% of encounters [20].

In summary, studies involving direct real‐time observation of communication within the diagnostic process are relatively limited. Even rarer is research that combines such observation with data collection from doctors *and* patients to understand their respective narratives: no such research has examined diagnostic processes in UK acute secondary care. We therefore conducted this ethnographic study, which had the broad research goal of characterising the processes through which differential and definitive diagnoses are made, communicated and documented. The theoretical basis for the study was informed by previous empirical work (e.g., a study examining the communication of information about possible diagnoses, which highlighted that patients want more information and are less concerned than physicians about potential negative effects of such communication) [38] and a review of ethical, philosophical and legal literature (investigating the relationship between communicating uncertainty, maintaining trust and balancing responsibility). Such ideas about diagnostic uncertainty and its communication served as sensitising concepts [39] that helped to guide the present study.

In this paper, we present the results of an analysis of the ethnographic data that focused on comparing doctor and patient perspectives to explore reasons for their divergence or convergence. We focus on identifying communicative practices that facilitated or inhibited shared understanding about diagnoses between patients and doctors.

## 2 Methods

### 2.1 Interdisciplinary Ethnographic Approach

Ethnography is ‘the study of social interactions, behaviours, and perceptions that occur within groups, teams, organisations, and communities’ [40]. It can be useful in gathering empirical insights into phenomena that are typically ‘hidden’ from the public gaze [40], and is often used in health services improvement research due to its ability to identify concealed conditions of risk [41, 42]. It is especially well‐suited to identifying the informal interactions that may create or prevent risk and shedding light on the multiple factors that shape clinical practice.

In this study, the ethnographic approach facilitated holistic in‐depth insights into diagnostic processes, by capturing snapshots of individual diagnostic journeys as they occurred within acute settings (including the social interactions, perceptions and communicative behaviours making up such processes). This approach allowed the examination of important aspects of clinical work that may be invisible or difficult to articulate by professionals themselves, and may not be amenable to measurement in the traditional sense.

Triangulation added depth: we used both data triangulation (using different sources of data to examine a phenomenon in different settings or points in time/space) and investigator triangulation (using multiple researchers to generate a range of perspectives) [40]. Our research team consisted of medical doctors and social scientists with backgrounds in anthropology and medical ethics, respectively. The range of different perspectives was important in facilitating an interdisciplinary approach—input from researchers from different academic disciplines allowed a better appreciation of the nuances of the data. Non‐clinicians were able to highlight practices that the clinicians failed to notice as anything other than ‘how things are normally done’, whereas the clinicians were able to provide clinical context to interactions, reading between the lines of what doctors were thinking by applying clinical reasoning to understand the nuances of what was said or unsaid.

### 2.2 Setting

Data were collected in acute medical sectors of three hospitals (emergency departments, acute medical and ambulatory care units) across England. Hospitals were purposively selected to ensure heterogeneity of practices was observed: they varied in size, geographical location, populations served and information infrastructure (fully electronic‐based vs. an electronic/paper‐based hybrid). Access was facilitated by senior acute medicine physicians in each hospital, who helped ensure that staff and patients were informed about the study. Posters were also displayed in patient‐ and doctor‐facing areas to notify participants.

### 2.3 Data Collection

Our ethnographic approach comprised of (1) observations and (2) semistructured interviews with doctors and patients. Data were collected by two social scientists with experience in conducting ethnography (T.H., J.W.) and one consultant physician in acute medicine (Z.F.); researchers worked independently.

Data were collected in two phases between July and August 2022 and between December 2022 and February 2023. Observations were initially open‐ended; in the second phase, the research team developed a guidance document to focus observations on areas to investigate further. The phased approach allowed analysis and debrief at the mid‐point, enabling refinement of our approach by identifying where to probe further in interviews.

There were four separate data streams:

1. *Observations*: Researchers purposively sampled doctors (to ensure inclusion of doctors with a range of demographic characteristics and clinical experiences). Researchers shadowed doctors as they assessed and initiated investigations/treatment for undifferentiated acute patient presentations (i.e., patients who did not yet have a confirmed diagnosis at this initial contact). Oral consent was obtained from participants. Researchers took contemporaneous written notes and audio‐recorded or typed reflections at the end of each day's observation, producing a set of written field notes for analysis. We observed for approximately 8 h a day, in 2‐ or 3‐day stints, accounting for an approximate total of 28.5 days (228 h) of observations equally spread across the sites. Observations took place on weekdays from early morning to late evening.
2. *Doctor interviews*: Researchers conducted semi‐structured interviews with 24 doctors who were recruited during the observations. These took place face to face in private rooms in the hospital or via telephone as soon as possible after the observed consultation. Interviews explored how they made, recorded and communicated the (differential) diagnosis. All interview participants signed a consent form; all interviews were audio‐recorded, transcribed and anonymised.
3. *Contemporaneous patient interviews*: Researchers conducted semi‐structured interviews with 32 patients; as with doctor interviews, these took place either face to face immediately after the observation or via telephone soon afterwards. Patient interviews explored what the patient understood about their diagnosis and how they felt about their experience.
4. *Follow‐up patient interviews*: Patients were invited to interview again 2–4 weeks after their first interview to see what they remembered about their diagnosis. 15 patients completed these follow‐up interviews via telephone.

See Appendix SA for all interview guides.

### 2.4 Data Analysis

**Figure 1:**
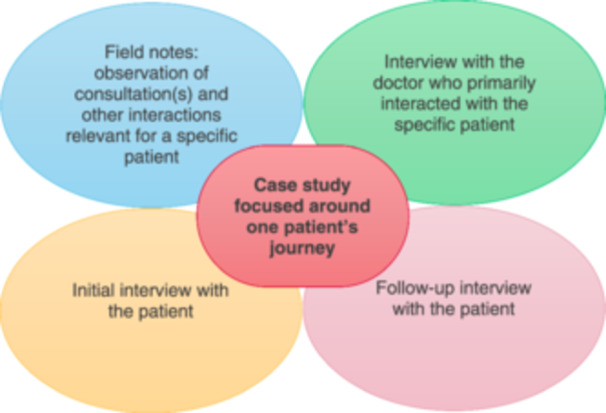
Case studies consisting of data from four streams.

We combined observation of a patient's initial assessment and treatment with both doctor and patient interviews to create ethnographic case studies (see Figure 1) [43]. Each case study permitted detailed examination of the concerned unit of analysis (here, a single patient's journey) within the acute care setting [44]. The ethnographic lens explored how diagnoses were formed and communicated, whereas the creation of case studies helped to bind the research in space and time around specific patient journeys [45]. These case studies facilitated the direct comparison of doctors' and patients' experiences of diagnosis, enabling researchers to use triangulation to identify patterns in how doctors communicated, and how patients understood, diagnoses.

In keeping with established ethnographic methods [46], throughout data collection, the research team conducted debriefs to facilitate reflexivity and synthesis. We took an iterative approach to analysis, drawing from the constant comparative method [47]. Case studies provided a basis for collaborative thematic analysis: members of the research all initially reviewed the case studies, before then undertaking an inductive open‐coding approach, gradually organising codes into broader themes. Constant comparison of data from different streams within each case study—and between case studies—facilitated within‐ and between‐narrative comparison; we particularly looked for instances where doctors and patient perspectives significantly diverged or converged. An in‐person analysis day, which consisted of further rounds of inductive coding, facilitated the emergence of more abstract theoretical categories. This was followed by their deductive application to the data to assess their significance and validity [48].

Following analysis, we undertook member checking [49] by returning to share findings with doctors in the acute medical departments, gathering responses using an anonymised online platform. This enabled us to check clinicians felt that analyses were fair and representative. A range of doctors attended these sessions; all felt that we had fairly represented their practice.

## 3 Results

Doctors demonstrated relatively consistent broad diagnostic approaches, but varied in their communication of diagnostic information to patients. Although some patients left the consultation with an understanding that aligned with their doctor's, this was not always the case: often, patients had misinterpreted the information provided, or, in the absence of information, had made incorrect assumptions. Notably, many doctors were seemingly unaware of these misunderstandings.

We were able to highlight communicative practices that seemed to facilitate, or inhibit, shared understanding between patient and doctor. We identified three major themes: (1) communicating what has been understood from the medical record, (2) sharing the thought process and diagnostic reasoning and (3) closing the loop and discharge communication.

Boxes 1, 2, 3 summarise cases that exemplify the identified communicative behaviours; below, we discuss some cases in more detail. To protect patient confidentiality, we have systemically fictionalised the cases by withholding gender and altering some clinical details (as suggested elsewhere) [50].

Box 1 **Communicating what has been understood from the medical record.**

**Table:** 

Examples of communication that facilitated shared understanding	Examples of communication that inhibited shared understanding
S3P305—Patient with multiple presentations with chest pain. The doctor explicitly explained that they had examined the notes from previous attendances and knew about what investigations had already been performed. The patient was reassured that the doctor had taken the time to look through the notes. S1P209—Patient presenting with hypertension, doctor clear in explicitly stating that they looked through her notes and were aware of previous admissions with the same problem.	S3P310—Patient was frustrated as they felt that the doctors had not reviewed the past medical history in the notes. Although the doctors were aware of their history of thyroid disease, this was not made clear to the patient; subsequently, the patient was dissatisfied by this perceived lack of attention. S1P206—Patient with a complex cardiac background, who felt at times that doctors had not read their notes properly and did not understand their case. S3P315—patient with a long history of anaemia (for which they had been extensively investigated). The patient stated that they felt the doctor was ‘starting from scratch’, despite the doctor actually spending 40 min reviewing the notes before the consultation.

Box 2 **Sharing the thought process and diagnostic reasoning.**

**Table:** 

Examples of communication that facilitated shared understanding	Examples of communication that inhibited shared understanding
S1P204—Doctor clearly explained the diagnoses that were considered (pulmonary embolus vs. asthma exacerbation). The patient was relieved that PE was being considered, as it had been mentioned by their GP; communication of information about the possible diagnoses being considered relieved some patient anxiety. S2P201—Doctor clearly explained the differential diagnosis (of chest infection vs. pulmonary embolus), which the patient was able to recall clearly in the follow‐up interview. The patient praised the doctor for being honest about the possible diagnoses, stating that they would rather be told outright what was going on. S3P305—Patient with chest pain who was very satisfied with the doctor's explanation of the possible causes of the pain and details leading to the clinical decisions. S2P101—Doctor clearly explained possible diagnoses (e.g., pulmonary embolus vs acute coronary syndrome), linking these differentials to tests being performed. The patient demonstrated a good understanding of these possible diagnoses and was happy to be discharged with a diagnosis of possible muscular pain after more serious diagnoses had been excluded. S1P209—Doctor provided a clear explanation of the possible causes of secondary hypertension, which was accurately recalled by the patient in the follow‐up interview.	S3P310—A CT was arranged for a patient with headache, but the reasons for the CT were never explicitly discussed; the patient made incorrect assumptions that this meant the doctor thought they had a brain tumour. The patient stated a desire to be told exactly why tests were being done. S2P215—Patient presented with back pain and numbness. The doctor did not explain the rationale for the examination (particularly the rectal examination), and the patient did not understand why no X‐ray of the back was performed. They were frustrated as they felt that their concerns were not being properly addressed. S2P206—The doctor had a clear differential diagnosis for a patient with shortness of breath, but it was not communicated to the patient. The patient felt that they had not been told enough about the diagnoses being considered and would have valued information about what the doctors were testing for. The patient was already concerned about the possibility of a pulmonary embolus; although the doctor was considering this diagnosis, they did not communicate this to the patient. S3P312—Doctor had a differential diagnosis in mind (cellulitis vs. DVT), but these possibilities were not clearly communicated to the patient or their family member. Although the patient was started on antibiotics for possible cellulitis, the family member was left with the impression that the doctors did not have any idea of the cause of the leg swelling.

Box 3 **Closing the loop and discharge communication.**

**Table:** 

Examples of communication that facilitated shared understanding	Examples of communication that inhibited shared understanding
S1P202—Patient with chest pain who was given good written discharge information; they explained that they like to know exactly what had been done. S1P204—Patient who liked having a written record of all the investigations to be able to potentially discuss with their own GP, even if they did not fully understand all the results. There were, however, still some problems: the patient was confused by the use of technical language (‘pleurisy’), which they felt had not been explained to them at the time of their consultation. S3P305—Patient with chest pain who very happy with written documentation that they received (including scan results), as they felt it facilitated better understanding of the medical situation.	S3P310—Patient with a headache, who was discharged after a normal CT head result. The patient was frustrated as they were discharged by a nurse rather than the original doctor who had organised the scan, and as such, did not have any opportunity to discuss the result with the doctor. S1P201—Patient with chest pain who was seen on discharge by a more junior doctor than the one who had done the initial assessment. The patient felt that this doctor was unable to properly answer questions about the cause of their pain or about the implications for an upcoming holiday. The patient then re‐presented to hospital the day after discharge as a result of ongoing unresolved questions about the diagnosis. S1P204—Patient with shortness of breath who did not feel they had space to ask questions about the diagnosis on discharge as they felt the doctor to be too rushed and kept cutting them off. S2P202—Patient who did not receive any discharge letter. They wished that they had been given written information, highlighting that they could not remember everything that they had been told. S1P206—The patient's discharge letter contained information that they did not recall ever being told or asking about.

### 3.1 Communicating What Has Been Understood From the Medical Record

Doctors spent up to 40 min reading patients' notes before consultations, gathering information and sometimes generating a preliminary differential diagnosis. However, doctors often gave patients no indication of having done this: patients neither saw their doctor reading their notes, nor did the doctor explain that this had happened. Consequently, patients often assumed that the doctor had not read their record.

The opening remarks of the consultation fuelled these assumptions. Opening questions such as ‘what brought you to the ED today?’ [S3P305] or ‘so tell me what happened’ [S2P210] gave the impression that the doctor was starting from a blank canvas. This contrasts with a minority of doctors who opened with an acknowledgement that they already had some background information, such as ‘I've looked at your notes, but how are you, what's happened?’ [S1209].

In interviews, doctors explained that they treat a patient's medical notes with an air of suspicion, to avoid other clinicians' interpretations clouding their clinical judgement. Describing why he asked patients about things that he had already seen on a record, one doctor explained:

[Y]ou just have to check … because if everyone just assumes that the first person has got it right and then it's wrong then mistakes happen … I've just learnt that you just have to be really sceptical.

[S1D201]

Although this may explain why doctors often avoided explicitly acknowledging what they have understood from the notes, patient reactions highlight unintended consequences of this practice. Several patients (particularly those with a complex history) were frustrated by a perceived failure of their doctors to properly look up their medical background:

They didn't know the last time I'd been in A&E … for the same thing. They didn't have any information about anything. And it was like, well, we're starting from scratch.

[S3P315]

I wasn't happy with was the fact that they didn't look … they don't tend to look at your record … they don't look at your history like they should. So I've got hypothyroidism … and I was trying to tell them … they went, oh, you've got thyroid problems. [they] should already know that because it's on the [notes].

[S3P310]

In both cases, the doctors had carefully reviewed the medical record and noted their patients' backgrounds. Not explaining what had been understood from the notes resulted in the patients thinking that their cases had not been properly reviewed, which contributed to some dissatisfaction and loss of trust.

In contrast, when doctors did explain what they already knew from reading the notes, this was perceived positively. A patient with multiple previous attendances with chest pain felt reassured when the doctor explained that they had looked through the notes and knew about her prior presentations and investigations:

I was happy when he said he's been looking at my previous records of chest pain, I felt he was really taking care of me, to go through all that with me.

[S3P305]

By discussing the information already gathered from review of the medical record, doctors can help build a shared understanding with patients, by helping them to appreciate what has already been done and what the doctor's current thinking is. This is particularly important when a patient is re‐presenting with the same symptom or if there is a particularly pertinent condition in the medical history.

### 3.2 Sharing the Thought Process and Diagnostic Reasoning

Doctors varied in the extent to which they shared their thought processes regarding the diagnosis (e.g., explaining what investigations were being arranged and why, or discussing the potential diagnoses being considered). When doctors did not communicate such information, it sometimes resulted in patient dissatisfaction; when doctors clearly communicated their thought processes, patients tended to have a better understanding of what was happening to them.

Frequently, patients made incorrect assumptions or were left confused when doctors did not explicitly explain reasons for examinations or investigations. A patient who presented with a headache (likely a migraine) had a CT scan, but the doctor did not explicitly discuss why the scan was being organised. The patient explained in an interview that this caused them to worry it could be a brain tumour.

I felt like I was left in the dark and I was questioning what's…wrong with me…I was thinking why do we need the CT, have I got a brain tumour, what's going on…

[S3P310]

Although the doctor had not mentioned a brain tumour, nobody explained what the CT scan *was* looking for. This patient expressed a desire to be ‘kept in the loop’ about the purpose of investigations:

I think they should be a little bit more in detail with … what they're looking for. Because I have found with a lot of doctors, they don't kind of keep you in the loop of what's going on, they just do their thing and then tell you the outcome at the end.

[S3P310]

We observed other examples of patients who made incorrect assumptions in the absence of clear information about why investigations were being performed. A patient with back numbness and pain was assessed by the doctor—including a full neurological examination, a bladder scan and a rectal examination (partly to rule out a dangerous differential, cauda equina). The examination was briefly explained as necessary to rule out an emergency, but no details were given about how this related to the patient's symptoms. In follow‐up interviews, the patient felt as though their primary concern (numbness in the back) had not been addressed. They did not understand why the doctor had focused so much on their limbs, bladder and rectum, rather than their spine:

I was very confused they didn't really examine my spine … I thought I was going to get an x‐ray… the main part where I can't feel in my body is my back, which she didn't examine, she only did my limbs, my … down there area, and the bladder … I can feel all that perfectly fine … I don't know why they checked my bladder, because I know it's okay … Just check my back, that's what I came here for … yeah, just frustrating.

[S2P215]

In both cases, patients did not receive a full explanation of why certain investigations were being carried out; they both left with a lack of shared understanding with their doctor about what was being looked for and residual questions about their condition.

Discussions about differential diagnoses were inconsistent; on some occasions, this communication was not clear, resulting in misunderstandings. For example, a failure to properly explain the diagnoses being considered meant that a patient with a swollen leg was left with the impression that the doctors had no idea about the cause for their symptoms—in fact, the doctor had a clear differential of cellulitis versus deep vein thrombosis and had commenced treatment to cover for both of these possibilities.

This pattern—of the doctor having a clear differential diagnosis in mind but not communicating it to the patient—was seen frequently. In another case, the doctor did not discuss any of the differential diagnoses (including pulmonary embolus [PE], chest infection and pleural effusion) with the patient. The patient was already concerned about the possibility of PE, as it had been mentioned by the paramedics; the patient stated in interviews that they felt that they were not properly told about the possible diagnoses and would have valued being given information about what was being tested for.

In contrast to this, a different patient with shortness of breath felt relieved when the doctor specifically discussed the possibility of PE, as this was a diagnosis their general practitioner had already mentioned to them. For this patient, greater explanation about the diagnoses being considered helped them to understand the diagnostic process and relieve anxiety around it:

Yeah, for me, it does [relieve anxiety], just because then I know exactly what they're looking for, so I know that the doctors have almost got a purpose, they're just not finding the needle in a haystack.

[S1P204]

Other patients similarly described how having the possible diagnoses and thought processes clearly explained helped to reduce anxiety and increase understanding:

I would like them to be honest with me, to tell me what…it could be, like they have done, rather than … keep me in suspense because I think you worry more if you don't know, whereas if you're told properly what it could be, then you are more prepared for whatever … I would rather be told outright what's going on.

[S2P201]

I felt alright because he gave me … the details leading to the decision, so I understand where he's coming from.

[S3P305]

That patients appreciate being given more information about the diagnostic process, including the diagnoses being considered and any related uncertainty, was noted by several doctors we interviewed:

[W[hat I've learnt the patients really appreciate is if you just … tell them the whole picture … … if I tell them everything that I know and explain where the uncertainty is … and why my diagnosis isn't concrete then they appreciate that, and then they…they're on the same page as me.

[S1P201]

In several cases, when doctors clearly explained the differential diagnosis, patients were able to accurately recall this information in follow‐up interviews, demonstrating shared understanding of diagnosis with their doctors.

Overall, we identified that failing to explain the reasons for doing investigations may lead patients to make inaccurate and anxiety‐inducing assumptions about their differential diagnosis, and to feel frustrated if they lack understanding about why investigations were being arranged. In contrast, clear explanations of diagnostic thought processes facilitated shared understanding between doctor and patient and often reduced anxiety.

### 3.3 Closing the Loop and Discharge Communication

A third area where we identified potential for misunderstanding was communication at the end of the consultation and on discharge. We noted several patients who were frustrated because they had no opportunity to discuss investigation results or their ongoing care before discharge, as they were discharged by a different member of staff to the original doctor they had seen. They left feeling that their care had been disjointed and incomplete as they were unable to ‘close the loop’.

An example was the patient who had presented with headache, for whom a CT scan was organised by the initial assessing doctor. When the CT scan was normal, they were given this information and discharged by a nurse rather than the doctor who arranged the scan. The patient was disappointed not to have had the opportunity to ask questions about the cause of the headache or how to prevent them in the future:

… a nurse coming up to me and saying CT's done, you're fine, you can go … that really frustrated me … Because obviously the CT scan was giving answers, so I was waiting that time to then speak to a doctor, and I wasn't given a chance.

[S3P310]

This patient was not alone in experiencing such frustrations: we observed several patients who were left dissatisfied when discharged by another member of staff without receiving closure or being able to ask questions to the doctor with whom they had built a rapport. A patient with chest pain was left disappointed on discharge by a discussion with a more junior member of staff (rather than the initial doctor they had seen):

she was quite abrupt … She said … you haven't had a heart attack but we are going to do a referral to you to the chest pain clinic … So I said, well, what about my holiday? She said, we can't make that decision for you. It's up to you … to decide whether you think you're well enough to go.

[S1P201]

As a direct result of these unresolved questions about the diagnosis, this patient re‐presented to hospital the next day, where they were admitted and underwent further investigations that were ultimately normal. In this case, if the patient's questions about the diagnosis had been properly addressed before discharge, they may not have had the second admission.

This opportunity to ask questions before discharge was particularly important when doctors and patients had differing expectations around diagnosis in the acute setting. We noted that doctors were often focused on ruling out the serious or life‐threatening conditions. In contrast, patients typically did not just want to be told that the symptoms were not caused by something life‐threatening, but rather what the diagnosis was or how their symptoms might be managed. When expectations about what was achievable in the acute setting were misaligned, it sometimes resulted in patients feeling as though the diagnostic process has ‘failed’ and they had not been properly listened to. Patients being discharged without a definitive diagnosis (such as the above patient with chest pain) were particularly vulnerable to feeling frustrated by the diagnostic process—and were more likely to be dissatisfied when not given the opportunity to ask questions before leaving.

The provision of written information on discharge was very inconsistent: although in interviews doctors stated that patients always get a written copy of their discharge summary, this was rarely the case. This lack of information contributed to some patients having an imperfect understanding of what their likely diagnosis was. As one patient explained in a follow‐up interview:

I kind of wish I had got a discharge letter … you can't remember everything. Then I was like oh what did they say, what [medication] do I take, is it liquid, is it tablets, do you know what I mean. I really wished I had a copy myself to know … what they thought it was.

[S2P202]

Even when patients did receive written information on discharge, sometimes, it caused confusion. One patient was happy to be able to review a written record of their admission on a mobile platform, but was confused by the use of technical terms (‘pleurisy’). Although the doctor had explained the concept to them in lay terms, the term ‘pleurisy’ had not been used; they were left feeling as though the doctor had not properly verbally explained the diagnosis or had even withheld information.

In some cases, patients did receive accurate written discharge information, which was helpful in facilitating a better understanding of the diagnostic process. For these patients, being given clear information helped them to better understand what had been done and why.

[Information] given to the patient that day, so they could read what [doctors] are actually talking about … That's like when you take your car into a garage and that, I always want to know … I just like to know what's going on and what's been replaced…when they do a car service.

[S1P202]

The difficulty in providing patients with discharge information that is understandable was reflected on by doctors:

Yes, a summary, even in medical terms is fine … but if it's going to be every bit of documentation of their healthcare, that's going to make them more anxious … that's a double‐edged sword.

[S2P212]

In summary, communication at and after discharge is important in developing a shared understanding between doctor and patient about what has happened during a hospital attendance. Failure to ‘close the loop’ can negatively impact on patient satisfaction, and the failure to provide clear written communication on discharge can leave patients with a poor understanding of the doctor's conclusions about the possible or likely diagnosis.

## 4 Discussion

By analysing doctor versus patient perspectives—and comparing them with researcher observations—we identified communicative behaviours that seemed to facilitate or inhibit shared understanding of the diagnostic process. This study provides insights into communication within undifferentiated diagnostic processes in a UK secondary care setting, about which there was limited existing empirical research. On the basis of our findings, we suggest practical recommendations for how doctors might communicate with patients to better foster shared understanding within the diagnostic process in acute settings (Box 4).

Box 4 **Recommendations for practice.**

**Table:** 

Doctors should tell their patients what they have read and understood from their medical record. Doctors should explain why certain tests have and have not been ordered. Doctors should discuss what diagnoses are being considered, particularly if investigations are being arranged to specifically rule them out or in. Doctors should make every effort to speak to patients themselves at the point of discharge to answer any remaining questions and check their understanding of the medical encounter; where they anticipate that this will not be possible, they should signpost who will be coming back and emphasise that questions can be asked to them. Patients should be provided with written information on discharge, explaining what investigations have been performed and what the likely diagnosis is (including information on any diagnostic uncertainty and any other important alternative diagnoses). Doctors should aim to establish diagnostic expectations for the specific setting.

We found that greater transparency about the diagnostic process often resulted in increased doctor–patient shared understanding and increased patient satisfaction. Although this may seem intuitive, the heterogeneity of practice observed suggests that clinicians currently interpret and act upon notions of transparency variably. Examples of communication practices that fostered shared understanding included when doctors shared information about what they had read in the medical record, what investigations were being arranged and why and what diagnoses were being considered. Conversely, when doctors did not explain their diagnostic thinking, patients were more inclined to make incorrect assumptions about the (differential) diagnosis or feel as though their concerns had not been addressed. Communication at discharge was important in giving patients the opportunity to clarify details and ask further questions (particularly for those being discharged without a definite diagnosis).

Our results are consistent with the broader sociological literature emphasising differences in patient versus doctor perspectives, which has explored the role of negotiation between contradictory illness models in building patient–doctor understanding [51, 52, 53, 54]. Such theoretical work is supported by some empirical studies, which have demonstrated that patients' differential diagnoses are often very dissimilar to their doctors' [17, 55] and that worries about possible diagnoses are commonly unvoiced in consultations [55, 56]. Our study's exploration of patient versus doctor perspectives adds to this work, reinforcing the idea that patients and doctors frequently have different ideas about possible diagnoses. In this context, it is unsurprising that if doctors do not explicitly share their thought processes—including explaining what diagnoses are being considered—inaccurate patient assumptions about possible causes for symptoms can persist.

One example of inaccurate assumptions occurs when doctors failed to explain what they had understood from the medical notes: patients often assumed that their record had not been properly reviewed. Although research has explored the impact of patients having access to their notes [57, 58, 59, 60], little has examined the effects of doctors (not) being explicit about what they have learned from the notes. Patients in the emergency department often have to retell their story to multiple doctors [11]. Our study adds to the existing literature by highlighting that if doctors do not make clear that they are aware of what others have already documented, patients can be left feeling frustrated by a perceived lack of attention. This suggests an important role for doctors communicating what has already been gleaned from the notes (or their colleagues) at the start of an encounter.

Another key finding of our study—that patients valued when doctors shared their diagnostic thought processes—is noteworthy, given the relative paucity of existing research examining patient preferences for communication throughout the diagnostic process. What patients want to be told regarding possible diagnoses (including any diagnostic uncertainty) has not been widely studied [61, 62]. A recent video vignette study explored patient communication preferences in hypothetical scenarios involving diagnostic uncertainty, finding that the majority of patients preferred greater communication of information relating to possible diagnoses and related uncertainty [63]. Participants appreciated when doctors shared their thought processes and discussed differential diagnoses, as such communication helped to foster greater understanding of their medical situation. The present study builds upon this vignette study by confirming that real (in addition to analogue) patients value such communication.

One area that has been studied more extensively is emergency department discharge communication [64, 65]. It has been widely demonstrated that patients often have a poor understanding of their diagnosis after discharge [12, 13, 14, 15, 16, 17, 18, 19]. Although many studies have reported these knowledge deficits, fewer have explored why they occur: some have found associations between patient demographic factors (e.g., age and educational attainment) and post‐discharge understanding [14, 15, 16, 66], but relatively little research has directly examined how communicative practices during the patient encounter may contribute to poor post‐discharge understanding.

Our study provides useful insights in this area. For example, we found that sometimes, the failure of many doctors to ‘close the loop’ by answering questions before discharge left patients with unresolved queries about their diagnosis. Other research has also demonstrated deficits in this sort of discharge communication: Musso et al. found that the treating physician failed to return to speak to the patient before discharge in 29% of encounters [20], whereas Rhodes et al. found that only 16% of patients were asked whether they had questions at discharge, with no instances of the doctor confirming patient understanding of the information [37]. Our study suggests that improving such communication may be helpful in improving shared doctor–patient understanding about diagnosis after discharge. There is a need to further evaluate different methods of delivering diagnostic information to patients and of methods to check their comprehension—for example, there is promising evidence that ‘teach‐back’ techniques can be helpful in improving patient understanding of information on discharge [67, 68, 69].

Finally, our study indicates potential deficits in current practices around providing patients with written discharge information. In our study, written information was rarely provided to patients, but patients suggested that it is helpful in improving understanding of the medical encounter. There have been some attempts to establish whether the modality of discharge communication (verbal vs. written vs. video) influences patient comprehension—the results are somewhat mixed, although several reviews have concluded that providing written and pictorial materials at discharge improves patient recall of information [65, 70]. The provision of a brief written discharge information card after ED attendance has been shown to increase patient awareness of the discharge diagnosis [71], suggesting that the written information does not have to be extensive to improve shared diagnostic understanding.

Future research must consider potential unintended consequences of increased communication throughout the diagnostic process, for example, the additional time pressure that it might place upon doctors. There is also a need to evaluate the impact of increased communication (during the encounter and at the point of discharge) on outcomes, including patient satisfaction, trust and healthcare‐seeking behaviours (e.g., adherence to follow‐up and likelihood of returning in the event of persistent or worsening symptoms). Medical education could have an important role in fostering changes in communication practices—including uncertainty communication [72, 73]—and a number of medical education interventions have been developed in the US focussing on diagnostic uncertainty communication [74, 75, 76, 77, 78].

## 5 Strengths and Limitations

Two major strengths of this study are its interdisciplinary nature and the triangulation of observations with interview data from doctors and patients. Rather than collecting data by retrospective review of notes or asking patients/doctors to recall details of previous encounters, we directly collected real‐time data. This is important, as other studies have demonstrated discrepancies between what patients remember of consultations and what researchers have observed [20]. Our study design permitted the collection of data relating to each patient encounter, facilitating direct comparison of doctor versus patient perspectives. We collected data over 228 h, observing physicians of a variety of grades in three different hospitals, increasing the generalisability of results.

Our study has several limitations. Participants may have changed their behaviour as a result of being observed, producing a Hawthorne effect—doctors may have provided more detailed communication compared with their normal practice because of social desirability bias. The fact that we still observed many instances of doctors not clearly communicating diagnostic information is therefore notable: it may be that in unobserved practice, such poor communication practices are more widespread.

Observations were only conducted during weekdays, from mornings to early evenings; there may be differences in communication at weekends or overnight, which our study was unable to detect. The interactions that we observed tended to be with patients with lower acuity/severity health problems—the generalisability of these results to patients presenting more severely unwell is therefore unclear. We purposively sampled doctors (to ensure inclusion of doctors with a full range of demographic characteristics and clinical experiences), but there may still be some selection bias—the doctors who agreed to participate (and the patients who agreed to be interviewed) may systematically differ compared with those who declined.

A disadvantage of using researcher observation (rather than audio/video recording consultations) is that the researchers inevitably introduce their own interpretation of events and their recording of interactions may be biased by their backgrounds and previous experiences. Investigator triangulation was used to mitigate against this.

## 6 Conclusion

This study found that in UK acute secondary settings, the provision of more information about the diagnostic process often fostered shared understanding between doctor and patient, helping to minimise the confusion and dissatisfaction that can result from misaligned expectations or conclusions about the diagnosis. Communication intended to make sure that doctors and patients were ‘on the same page’ was effective in facilitating a shared understanding of the diagnostic process. We have made some practical recommendations for how doctors working in acute settings might improve their communication to improve patient understanding and have suggested areas for further research to evaluate whether these changes in communication may also impact patient outcomes.

## Author Contributions

**Caitríona Cox:** writing–original draft, writing–review and editing, formal analysis. **Thea Hatfield:** investigation, writing–review and editing, formal analysis. **Janet Willars:** writing–review and editing, formal analysis, investigation. **Zoë Fritz:** conceptualisation, methodology, investigation, funding acquisition, writing–review and editing, formal analysis, supervision.

## Conflicts of Interest

The authors declare no conflicts of interest.

## Supporting information

Supporting information.

## Data Availability

The participants of this study did not give written consent for their data to be shared publicly, only that the anonymised data collected may be used to support other researchers in the future. As such, the data that support the findings of this study are only available on request from the corresponding author, C.C.
