# Supplementary material for: Identifying Facilitators and Inhibitors of Shared Understanding: An Ethnography of Diagnosis Communication in Acute Medical Settings
Source: Health Expect. 2024 Aug 24;27(4):e14180. doi: 10.1111/hex.14180 (PMC11344224; doi:10.1111/hex.14180)
Supplement: Supplementary file 1 — Supporting information. [file HEX-27-e14180-s001.docx]

**Semi structured interview for Patients and/or their relatives**

*These interviews will take place after they have seen a clinician in the acute setting. They are designed to explore their experiences of communication of their (differential) diagnosis and their beliefs about information sharing, responsibility and patient trust.*

*We are interested in finding out about what happened from when you realised you needed medical attention to now*

1. Please can you tell us what happened, and who you spoke to. (Ask questions as narrative is relayed if more detail needed).
2. What did the doctor you saw most recently tell you? (if not volunteered: “Did you feel that you were told about your diagnosis or possible diagnosis?”)
3. Do you know what the next steps are for you?
4. Did you have any suspicions of what might be going on before you saw the doctor? what made you worried about this?

*[If tests/ treatments planned..]:*

1. Did the doctor in the acute/ emergency department explain why they were doing those things?
2. Did they give you any choices/ information about what would happen next? (in next hours, days and weeks)

*To all patients:*

1. Was there anything else they explained?
2. Is there anything you are wondering about now?
3. Did you have the chance to ask questions?

*If* ***yes*** *to question 8:*

1. (a) What questions did you ask?
2. (a) Were the questions answered to your satisfaction?
3. (a) Was there anything else you would have liked them to tell you/explain?

*If* ***no*** *to question 8:*

1. (b) Why do you think that was the case?
2. (b) Was there anything you would have liked them to tell you/ explain?
3. Did you feel you had enough time to say everything you wanted to say?
4. Did you feel involved in the decisions? How did you feel about this?
5. Did you feel you could trust the doctor you just saw?
6. Can you tell me why?/why not?

*[if not prompted by what they have already said]:*

1. did they tell you what they thought might be wrong with you?

*[depending on what was said]*:

1. How certain did you think your Dr was about the cause of your symptoms ( your diagnosis)?

1. How would you have felt/ did you feel if the doctor had said they weren’t sure about what the cause of your symptoms was?
2. [*if not given a definitive diagnosis*] did you come in expecting a diagnosis?
3. *[if been told that sinister conditions ruled* *out*] are you satisfied with the level of investigation/ leaving without a clear diagnosis?

*[If being discharged]:*

1. Did you speak to a doctor about being discharged?
2. Did they give you any information about what they thought caused your symptoms/ under what circumstances to come back?

*[IF patient has been given some responsibility e.g. on needing to come back if they get sick again, a delayed prescription, or some choice in whether to have investigation]*

1. How did you feel about that?
2. Do you think it is reasonable for the doctor to share the responsibility of making that decision with you?
3. Why? / Why not?
4. Did it affect your trust in the doctor?
5. Did you see your doctor writing any notes? what do you think they wrote? Do you know where that information goes?

*IN general - sometimes after taking your history a doctor may suspect that you may be suffering from one of two/three different complaints. This is called a differential diagnosis…*

1. If you were teaching medical students, what would you tell them to do in this kind of situation?
2. If you were writing guidance on how information was shared with patients in an A&E/ Ambulatory Care unit/ AMU what would you recommend?

**FOLLOW up phone interview 2 weeks later**

*We are interested in what your thoughts have been since leaving hospital.*

1. Do you remember what the doctor said your diagnosis was?
2. Do you remember what tests were carried out/ what they were for?
3. Was the doctor you spoke to in hospital confident about this diagnosis?
4. Did they tell you to come back in any circumstances – if so do you remember what?
5. If memories are unclear- What do you think could have been done differently to help you better remember/ understand the doctor’s advice/ explanation?
6. Did they give you any written information/ did you receive any letters about your attendance/ diagnosis?
7. Have you thought of any questions since you left? – what were they?
8. Have you had any worry since you left? – what were they?
9. Are you thinking of going to see a doctor about the same problem?
10. Was there anything that happened in your care that you wish had not happened?
11. In general, are you happy with the care you received?
12. Is there anything you think was not communicated as well as you would have liked it to be?
13. Is there anything you think was communicated well?
14. Is there anything else that comes to mind that you wish to tell me?

**Semi-structured interview for Diagnosing Diagnosis for clinicians**

***Forming and communicating a Differential Diagnosis***

1. Can you tell me something about your thought process for the last patient you saw?

*(****prompt questions****: did you have a working diagnosis from before you saw the patient just from looking at the notes? the beginning? How did you form that? What else was on your differential? After speaking to the patient what was on your differential diagnosis and how did you form this? What things did you reject from your differential diagnosis? Why did you reject those?)*

1. How much of this thought process did you relay to the patient? (explore reasons why)
2. Were you certain about the diagnosis?
3. What do you think the doctors’ professional responsibilities are in terms of dealing with uncertainty? *(responsibility for anxiety, responsibility for thinking of all of the options, etc?)*
4. Did you relay uncertainty (if it existed) to the patient? How?
5. Why did you decide to tell/ not tell certain things?
6. Did you organise any tests? Why? What did you explain to the patient?
7. Did you start any treatments?

*(IF ‘just in case’ or while waiting for results - e.g. giving Dalteparin while waiting for a CTPA, or giving antibiotics when not clear they have a bacterial infection)*

1. Did you explain your reasoning for giving these treatments to the patient?
2. How would you describe the value or purpose of a diagnosis?
3. Do you think doctors and patients understand diagnoses in the same way?
4. Do you ever not reach a diagnosis? How do you feel about that?
5. Do you know anything about legal requirements or professional guidance to inform patients about uncertainty in diagnosis or alternative courses of investigation/ treatments?
6. Have you heard of the Montogmery case? Does this/ other case law impact your daily practice?
7. Have you/ do you ever seek guidance regarding if and how you should communicate uncertainty in a diagnosis/ alternate investigations with a patient? (for example, GMC?)
8. If you were teaching medical students, what would you tell them to do (in respect to communication, investigations, follow up) in this kind of situation?
9. If you were designing how information was shared with patients in an A&E / Ambulatory Care unit/ AMU what would you recommend?

***Recording a Differential Diagnosis***

1. What of the above process did you write in the notes? ( e.g. the differential diagnosis, the reasons for excluding some diagnoses, etc)
2. Did you have to ‘code’ the admission?
3. Was it clear how the admission should be coded?
4. What code did you give?
5. Would you have liked to put anything else? – what stopped you?
6. Would you have liked to put in any qualifiers? ( e.g. probable/ defined/ etc)
7. Did you add anything to a ‘problem list? ( often a record of the patients past medical history and active problems which can be seen across admissions)
8. Do you know where this will go, or how long it will stay on the records?
9. Do you see any problems/ advantages to this?

Can you tell me what institutional, policy or management factors you think influence how you communicate and record a differential diagnosis?
